# The Safety and Immunogenicity of the mRNA-BNT162b2 SARS-CoV-2 Vaccine in Hemodialysis Patients

**DOI:** 10.3389/fimmu.2021.704773

**Published:** 2021-06-16

**Authors:** Emanuel Zitt, Tamara Davidovic, Judith Schimpf, Armin Abbassi-Nik, Beatrix Mutschlechner, Hanno Ulmer, Magdalena A. Benda, Hannelore Sprenger-Mähr, Thomas Winder, Karl Lhotta

**Affiliations:** ^1^ Department of Internal Medicine 3 (Nephrology and Dialysis), Feldkirch Academic Teaching Hospital, Feldkirch, Austria; ^2^ Vorarlberg Institute for Vascular Investigation and Treatment (VIVIT), Feldkirch, Austria; ^3^ Agency for Preventive and Social Medicine (aks), Bregenz, Austria; ^4^ Department of Internal Medicine 1, Feldkirch Academic Teaching Hospital, Feldkirch, Austria; ^5^ Department of Medical Statistics, Informatics and Health Economics, Innsbruck Medical University, Innsbruck, Austria; ^6^ Department of Internal Medicine 2, Feldkirch Academic Teaching Hospital, Feldkirch, Austria

**Keywords:** BNT162b2, COVID-19, hemodialysis, mRNA vaccine, SARS-CoV-2

## Abstract

**Background:**

Hemodialysis patients are at high risk for severe COVID-19. SARS-CoV-2 vaccination related safety and immunogenicity data in these patients are rare.

**Methods:**

In this observational study SARS-CoV-2-seronegative hemodialysis patients were vaccinated with two doses of the Pfizer/BioNTech mRNA-BNT162b2 vaccine (COMIRNATY^®^ 30 µg) and followed for 90 days. Local and systemic side effects were assessed at every dialysis session during the first post-vaccination week after the first and second vaccine dose. Immunogenicity was determined four weeks after vaccination by quantifying anti-SARS-CoV-2 spike protein IgG antibodies (LIAISON^®^ SARS-CoV-2-TrimericS IgG chemiluminescent immunoassay) expressed in binding activity units per milliliter (BAU/mL) adapted to the WHO International standard.

**Results:**

Fifty patients (32% women, 68% men) with a mean (SD) age of 67.6 (14.8) years were included. Mild local reactions occurred in 38% after the first injection, and in 29.2% with mild, in 2.1% with moderate and in 2.1% with severe degree after the second injection. Systemic reactive events occurred less often, with diarrhea (4% mild, 4% moderate) and fatigue (8% mild) being the most frequent ones. After the first injection 42% of the patients developed a positive response using the assay specific cut-off value of 33.8 binding activity units per milliliter (BAU/mL) with a median (Q1, Q3) anti-SARS-CoV-2 spike IgG concentration of 20.0 (11.7, 51.0) BAU/mL. After the second injection the percentage of seropositive patients increased to 97.9% with an anti-SARS-CoV-2 spike IgG concentration of 1075 (290.8, 1735) BAU/mL. Higher age and immunosuppression were associated with lower, calcitriol treatment and prior seroconversion to hepatitis B vaccination with significantly higher antibody concentration.

**Conclusions:**

The mRNA-BNT162b2 SARS-CoV-2 vaccine appears to be safe and well-tolerated and shows a high immunogenicity in hemodialysis patients.

## Introduction

Patients with chronic kidney disease, and particularly hemodialysis patients carry a high burden of coronavirus disease 2019 (COVID-19) and are at highest risk for a severe course and death (1–3). The vast majority of these vulnerable patients are treated with in-center hemodialysis. This translates into an unpreventably increased risk of exposure to COVID-19 due to the frequent contacts with potentially infected patients, health care professionals or transport personnel. Several SARS-CoV-2 vaccines have proven to be highly effective to prevent COVID-19 in the general population (4–6). Hemodialysis patients were not included in the pivotal trials but have shown a similar seroconversion rate after SARS-CoV-2 infection as compared to the general population (7). Therefore, it seems plausible to assume an adequate seroconversion after vaccination. On the other hand, vaccination hypo-responsiveness has been shown in dialysis patients. As an example, the seroconversion rate after active hepatitis B vaccination is only 40-70% compared to >95% in healthy controls despite the use of a high-dose vaccine (8–11). Different host factors contribute to this impaired vaccination response, including age, presence of diabetes, an altered innate and adaptive immune response, nutritional status and vaccine characteristics such as formulation, dosage and administration route (12, 13).

The Austrian government and the Austrian National vaccination committee prioritized hemodialysis patients in the national vaccination strategy. We therefore were able to assess the safety and immunogenicity after a complete vaccination course using the mRNA-BNT162b2 SARS-CoV-2 vaccine in chronic hemodialysis patients.

## Patients and Methods

**Figure 1:**
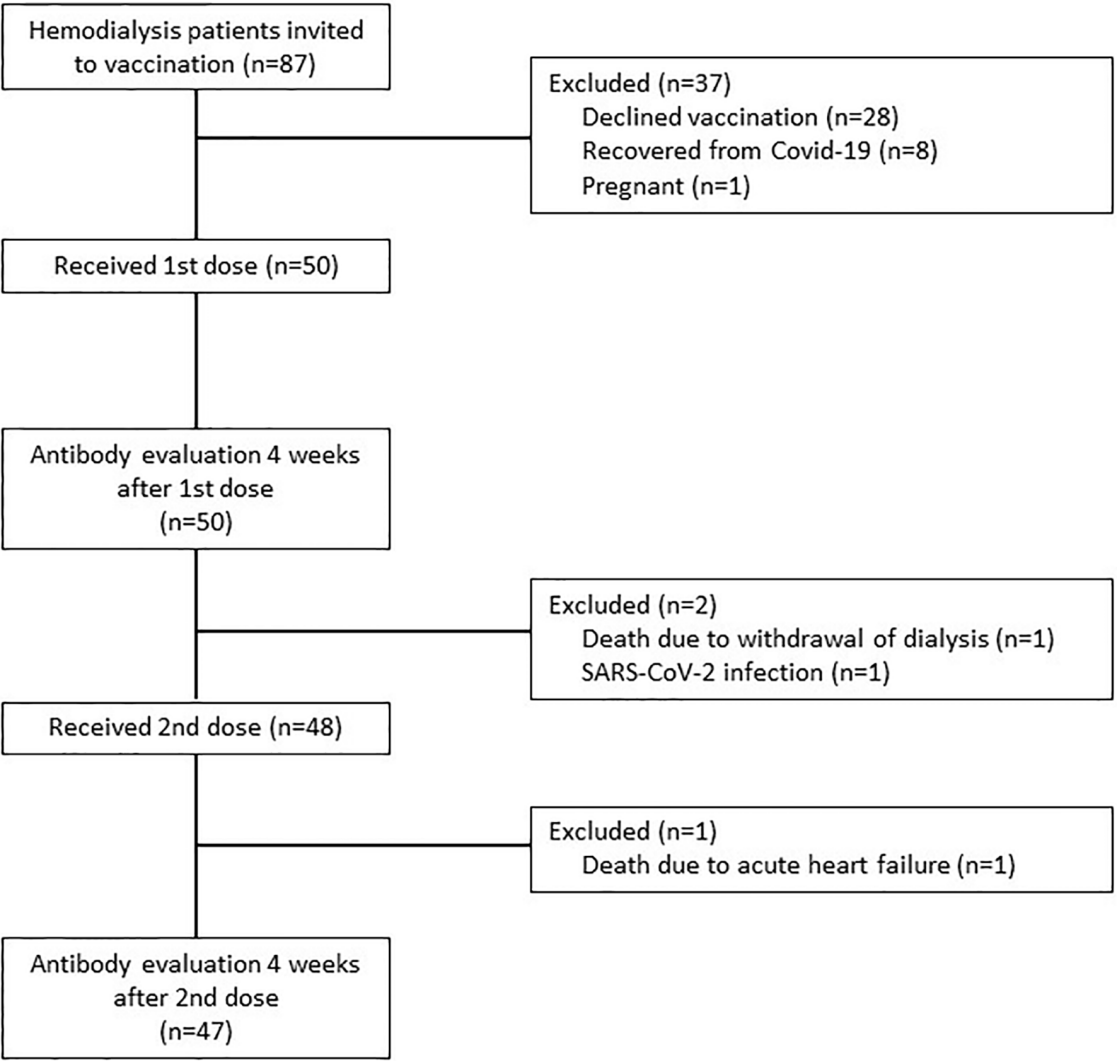
Patient flow diagram.

All chronic in-center hemodialysis patients treated at Feldkirch Academic Teaching Hospital, Austria, were invited to receive the SARS-CoV-2 vaccination following the prioritization by the National Vaccination Committee (14). Only patients with a negative anti SARS-CoV-2-serology were included in accordance with the National Vaccination Recommendation. After written informed consent all patients were vaccinated with the Pfizer/BioNTech mRNA-BNT162b2 SARS-CoV-2 vaccine (COMIRNATY^®^) with a dosing interval of 25 to 26 days between the first and second injection. The first doses were given on January 9^th^ and 11^th^, the second doses on February 4^th^ and 5^th^ 2021. Out of 87 hemodialysis patients, 50 received the first dose, and 48 completed the vaccination course with a second dose. A detailed patient flow chart is presented in **Figure 1**. Every patient received 30 µg of the vaccine delivered in the deltoid muscle of the non-fistula carrying arm using a 22 gauge (0.7 x 30 mm) needle (BD Eclipse^™^ Needle) approximately 30 minutes before the end of the dialysis session and was carefully monitored thereafter. Dialysis settings remained unchanged during the sessions the patients were vaccinated. No changes were made to anticoagulation. Low-molecular weight heparin was used as usual without dose adjustments, and patients on oral anticoagulation were not asked to reduce or pause oral anticoagulation therapy on the vaccination day. Baseline laboratory parameters were collected during the week prior to each vaccination as routinely recorded at the beginning of the month. Single-pool Kt/V was calculated using the Daugirdas formula (15) and averaged for one week.

All vaccinated patients were asked in personal interviews at each hemodialysis session during the first post-vaccination week for the occurrence of local and systemic side effects and reactions after the first and second vaccine dose. Body temperature was measured at the beginning of each session. Side effects and reactions were evaluated in accordance with the pivotal study of Polack et al. (5)

Immunogenicity was determined after the first injection on the day of the second injection and four weeks after the second vaccine dose by quantifying IgG antibodies from the patients´ serum. We used the LIAISON^®^ SARS-CoV-2-TrimericS IgG chemiluminescent immunoassay (Diasorin S.p.A., Saluggia, Italy). The assay detects IgG antibodies against the trimeric spike glycoprotein including the receptor-binding domain (RBD) and the N-terminal domain (NTD) sites from the S1 subunit. The assay has a clinical sensitivity of 98.7%, a specificity of 99.5% (95% confidence interval [95% CI] 99.0% - 99.7%), and has a very good correlation with the microneutralization test with a positive predictive agreement of 100% (95% CI 97.8% - 100%) and a negative predictive agreement of 96.9% (95% CI 92.9% - 98.7%). Test results were adapted to the WHO International standard for anti-SARS-CoV-2 immunoglobulin binding activity and presented in binding activity units per milliliter (BAU/mL) following the conversion equation “AU/mL*2.6 = BAU/mL” according to the manufacturer. A value of ≥33.8 BAU/mL was considered as evidence of a positive vaccination antibody response with seroconversion. The assay range according to the manufacturer is 4.81 - 2080 BAU/ml. For the detection of SARS-CoV-2- infection a commercially available RT-PCR (Seegene Allplex™ SARS-CoV-2 assay, Seegene Inc., Seoul, South Korea) targeting the three viral genes *E-, RdRP-* and *N-gene* on a nasopharyngeal swab was used.

This observational cohort study was conducted in compliance with the Helsinki Declaration of 1975, as revised in 2013, and Good Clinical Practice. The study protocol was approved by the institutional review board and the ethics committees of the Innsbruck Medical University (EK Nr: 1088/2021). STrengthening the Reporting of OBservational studies in Epidemiology (STROBE) guidelines were followed for the preparation of this article (16).

### Statistical Analyses

Categorical data are presented as absolute and relative number of patients. For continuous data mean and standard deviation (SD) or median with interquartile range (1^st^ quartile, 3^rd^ quartile) was used, depending on its distribution. Categorical parameters were compared using exact Chi-squared tests, normally distributed continuous parameters were analysed with Student’s T test and not normally distributed parameters with Mann-Whitney U test. A multiple linear regression analysis including the explanatory variables age, gender, dialysis vintage, diabetes mellitus, immunosuppression and calcitriol treatment was used to determine significant predictors of the antibody concentration four weeks after the second vaccine dose. The selection of explanatory variables for the multiple linear regression model was based on clinical importance and published literature, and restricted due to the patient number. The *R*² for the overall model was 0.314 (adjusted *R*² = 0.214), indicative for a high goodness-of-fit. A two-sided P value <0.05 was deemed to indicate statistical significance. All statistical analyses were performed with IBM SPSS Statistics 26 (IBM, Armonk (NY), USA).

## Results

Out of our total in-center hemodialysis cohort of 87 patients, 50 patients (32% women, 68% men) with a mean (SD) age of 67.6 (14.8) years received the first vaccine dose. As shown in **Figure 1**, 28 patients declined vaccination due to personal reasons, eight patients had recovered from prior COVID-19 with detectable antibodies and were therefore not prioritized for early vaccination, and one patient was pregnant and therefore excluded from vaccination. Forty-eight patients received the second dose, and 47 patients were available for the assessment of antibody response four weeks after the complete vaccination course. Safety data could be collected from 50 patients after the first injection and from 48 patients after the second dose. The baseline characteristics of all vaccinated patients are presented in **Table 1**.

**Table 1:** Baseline characteristics of the study population.

	n = 50
Gender, n (%)	
Female	16 (32.0)
Male	34 (68.0)
Age (years), mean (SD)	67.6 (14.8)
Dialysis vintage (months), median (Q1, Q3)	32.5 (17.8, 58.3)
Renal disease, n (%)	
Hypertensive kidney disease	13 (26.0)
Diabetic kidney disease	9 (18.0)
Glomerulonephritis	13 (26.0)
Other	15 (30.0)
Vascular access	
Arteriovenous fistula, n (%)	35 (70.0)
Arteriovenous graft, n (%)	3 (6.0)
Central venous catheter, n (%)	12 (24.0)
spKt/V^#^, mean (SD)	1.54 (0.24)
Diabetes mellitus, n (%)	13 (26.0)
Oral anticoagulation n (%)	8 (16.0)
Albumin (g/dL), mean (SD)	4.0 (0.5)
CRP (mg/dL), mean (SD)	1.0 (1.2)
Hemoglobin (g/dL), mean (SD)	11.5 (1.5)
Calcium (mmol/L), mean (SD)	2.11 (0.17)
Phosphorus (mmol/L), mean (SD)	1.84 (0.49)
PTH (pg/mL), mean (SD)	306 (171)
25(OH)vitamin D (µg/L), mean (SD)	19 (16.2)
Calcitriol supplementation, n (%)	33 (66.0)
Hepatitis B vaccination seroconversion^*^, n (%)	23 (46.0)
Immunosuppressive medication, n (%)	9 (18.0)
Glucocorticoid, n (%)	8 (16.0)
Tacrolimus, n (%)	1 (2.0)
Azathioprine, n (%)	1 (2.0)
Prior kidney transplant, n (%)	7 (14.0)

spKt/V, single-pool Kt/V; CRP, C-reactive protein; PTH, parathyroid hormone.

^#^spKt/V given as the weekly mean in the week prior to vaccination.

^*^Hepatitis B vaccination seroconversion defined by an anti-HBs antibody concentration ≥10 IU/L; n=13 patients with documented immunity after prior infection (positive anti-HBs and anti-HBc antibodies).

### Safety

Overall, the mRNA-BNT162b2 SARS-CoV-2 vaccine was well tolerated. Pain at the injection site within seven days after the injection was the most commonly reported local reaction, occurring in 38% of the patients of mild degree after the first injection. After the second injection, 29.2% of the patients reported mild, 2.1% moderate and 2.1% severe local pain. Despite regular blood circuit anticoagulation with low-molecular weight heparin and continuation of oral anticoagulation no hematoma occurred at the injection site, neither after the first nor the second vaccination.

**Figure 2:**
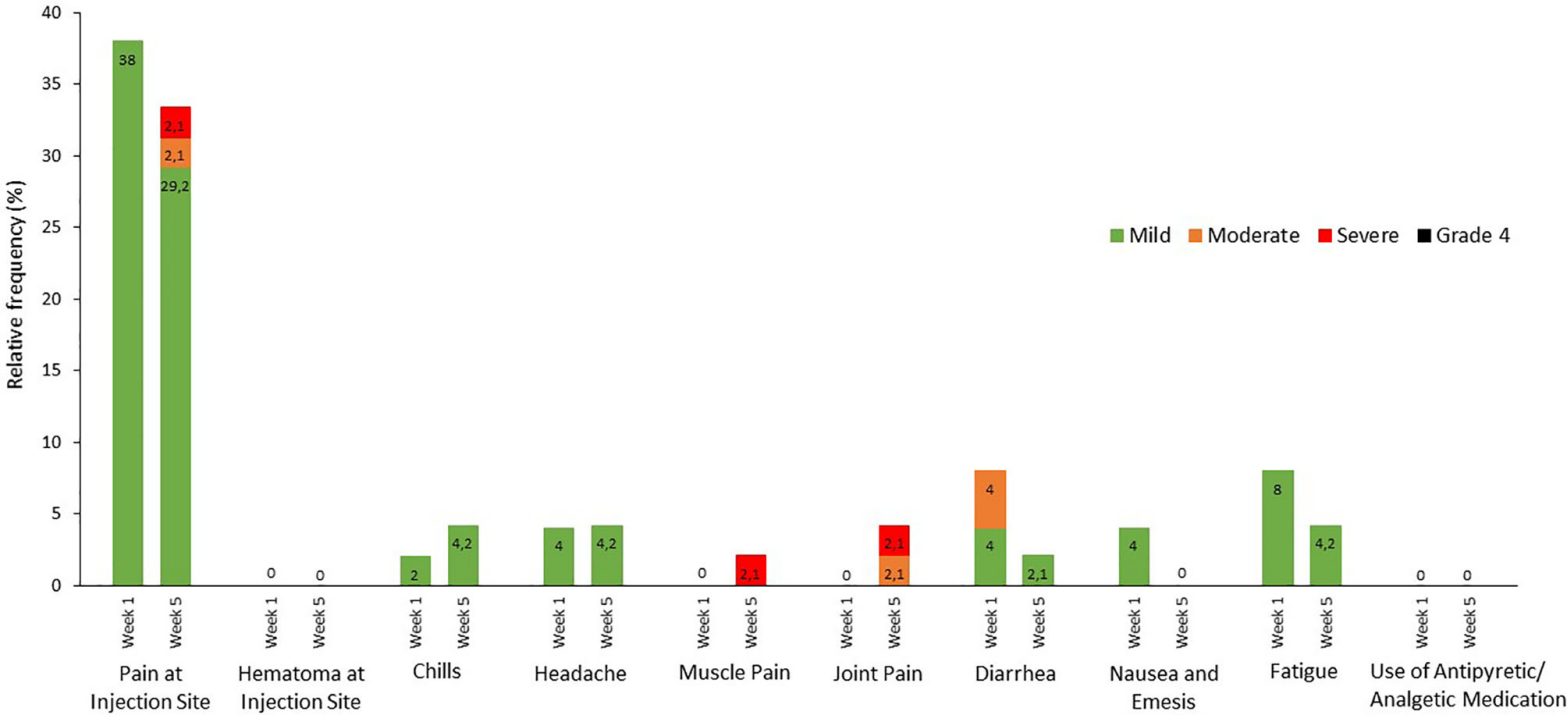
Local and systemic reactions occurring within seven days after vaccination. Data on local and systemic reactions and the use of antipyretic/analgetic medication were collected during each of the three hemodialysis sessions in the first week following vaccination. Pain and hematoma at the injection site was assessed according to the following scale: mild, does not interfere with daily activity; moderate, interferes with daily activity; severe, prevents daily activity; and grade 4, emergency department visit or hospitalization. The systemic reactions were graded according to the following scale: chills, headache, muscle pain, joint pain, fatigue: mild, does not interfere with daily activity; moderate, some interference with daily activity; severe, prevents daily activity; diarrhea: mild, 2 to 3 loose stools in 24 hours; moderate, 4 to 5 loose stools in 24 hours; severe, 6 or more loose stools in 24 hours, nausea & emesis: mild, vomiting 1 to 2 times in 24 hours; moderate, >2 times in 24 hours; severe, requires intravenous hydration; and grade 4 for all events indicated an emergency department visit or hospitalization. Medication use was not graded. Numbers in the bars are the percentage of participants who reported the specified reaction.

Systemic reactive events occurred less often, with diarrhea (4% mild, 4% moderate) and fatigue (8% mild) being the most frequent ones after the first injection. The occurrence of chills, muscle and joint pain slightly increased after the second injection, affecting 4.2% of the patients. None of the patients reported fever. In none of the patients a body temperature >38°C was measured before the start of dialysis at the three dialysis sessions during the first week after the injections. An overview of all local and systemic reactions within seven days after the vaccination is given in **Figure 2**. Two patients died during the study period. One patient deceased five weeks after the first injection because hemodialysis was discontinued and replaced by palliative care. Therefore, this patient did not receive the second vaccine dose. The second patient died from acute on chronic heart failure five days after the second injection. Both deaths were not considered to be related to vaccination.

### Immunogenicity

**Figure 3:**
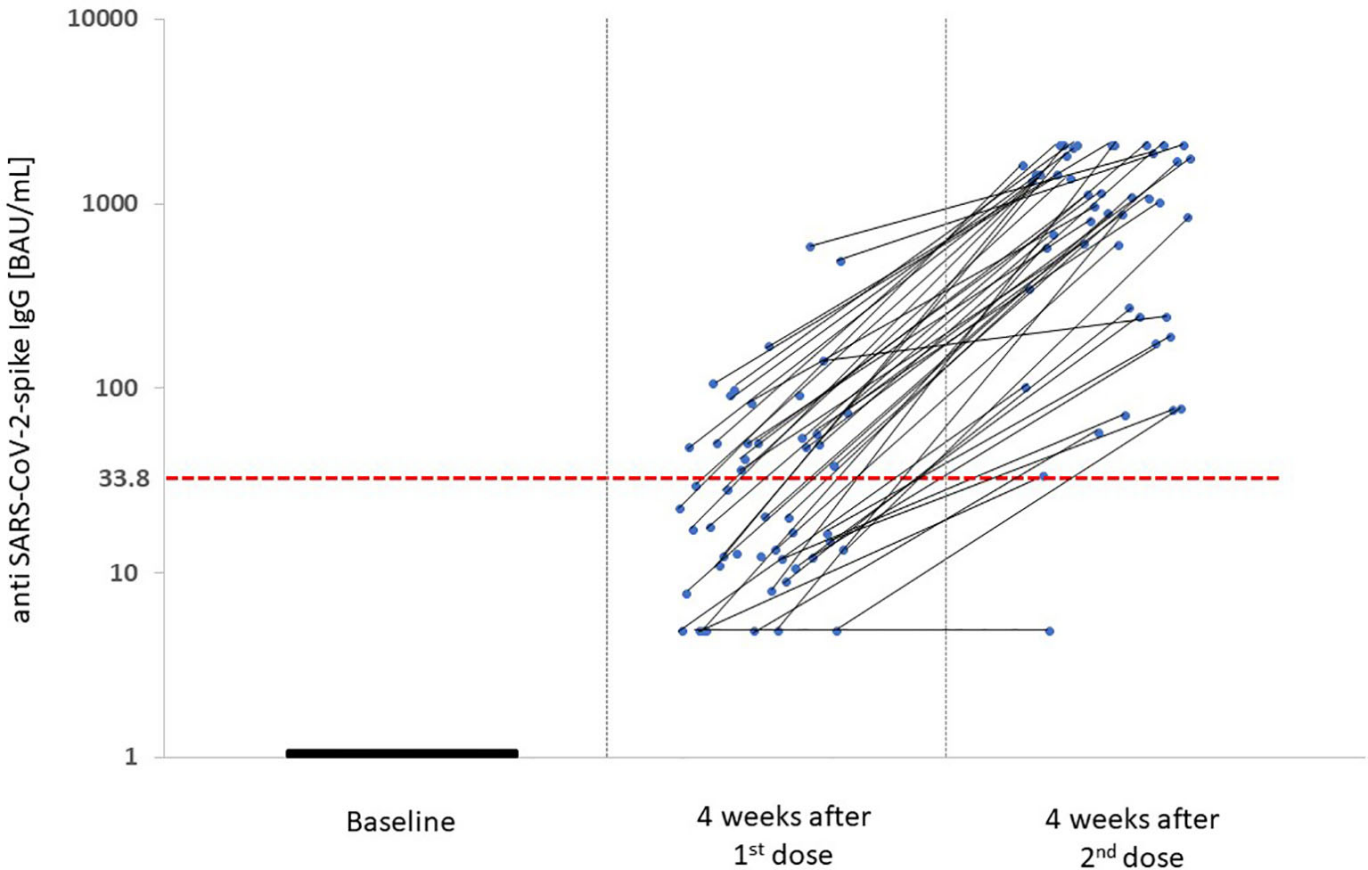
Anti-SARS-CoV-2-spike IgG concentration four weeks after both vaccine doses. Individual antibody kinetics after both vaccine doses are shown. Median (Q1, Q3) anti-SARS-CoV-2-spike IgG concentration four weeks after the 1^st^ vaccine dose was 20.0 (11.7, 51.0) BAU/mL, four weeks after the 2^nd^ vaccine dose 1075 (290.8, 1735.0) BAU/mL. The anti-SARS-CoV-2-spike IgG concentration is given in binding antibody units (BAU)/mL of the WHO International standard in log scale on the y-axis. The red dashed line presents the cut-off value for seropositivity in the Liaison^®^ SARS-CoV-2-TrimericS IgG assay of 33.8 BAU/mL.

Prior to vaccination, all patients were tested to be seronegative for anti-SARS-CoV-2 spike protein IgG antibodies. Four weeks after the first injection 42% of the patients developed a positive antibody response according to the assay specific cut-off value of 33.8 BAU/mL. The median (Q1, Q3) anti-SARS-CoV-2 spike IgG concentration was 20.0 (11.7, 51.0) BAU/mL. Four weeks after the second injection the percentage of seropositive patients increased to 97.9% with a median (Q1, Q3) anti-SARS-CoV-2 spike IgG concentration of 1075 (290.8, 1735) BAU/mL (**Figure 3**). Only one patient suffering from membranoproliferative glomerulonephritis type I with long-term corticosteroid therapy (ongoing low-dose regimen during the vaccination course) did not respond to vaccination (anti-SARS-CoV-2 spike IgG concentration 4.81 BAU/mL after the first and second injection). Patients with seroconversion after the first vaccine dose were significantly younger than those without (62.7 *vs* 71.2 years, p=0.043). Patient age was the only baseline characteristic that significantly differed between these two groups (**Table 2**). Patients with seroconversion after the first vaccine dose (56.7 [48.9, 101.7] BAU/mL) had a significantly higher median [Q1, Q3] anti-SARS-CoV-2 spike IgG concentration after the second dose than those without seroconversion after the first injection (1565.0 [1022.5, 2080.0] BAU/mL *vs* 635.5 [118.3, 1352.5] BAU/mL; p=0.001).

**Table 2:** Characteristics of patients with and without seroconversion after the 1^st^ vaccine dose.

Characteristic	Seroconversion (n = 21)	No seroconversion (n = 29)	P
Gender, n (%)			0.863
Female	7 (33.0)	9 (31.0)	
Male	14 (67.0)	20 (69.0)	
Age (years), mean (SD)	62.7 (16.1)	71.2 (12.9)	0.043
Dialysis vintage (months), median (Q1, Q3)	38.0 (11.5, 65.5)	31.0 (21.0, 52.5)	0.852
Renal disease, n (%)			0.357
Hypertensive kidney disease	3 (14.3)	10 (34.5)	
Diabetic kidney disease	5 (23.8)	4 (13.8)	
Glomerulonephritis	7 (33.3)	6 (20.7)	
Other	6 (28.6)	9 (31.0)	
Vascular access			0.885
Arteriovenous fistula, n (%)	14 (66.7)	21 (72.4)	
Arteriovenous graft, n (%)	1 (4.8)	2 (6.9)	
Central venous catheter, n (%)	6 (28.6)	6 (20.7)	
spKt/V^#^, mean (SD)	1.57 (0.27)	1.52 (0.23)	0.520
Diabetes mellitus, n (%)	6 (28.6)	7 (24.1)	0.724
Albumin (g/dL), mean (SD)	4.0 (0.4)	3.9 (0.6)	0.600
CRP (mg/dL), mean (SD)	1.0 (1.3)	0.9 (1.2)	0.867
Hemoglobin (g/dL), mean (SD)	11.5 (1.3)	11.6 (1.7)	0.784
Calcium (mmol/L), mean (SD)	2.09 (0.17)	2.12 (0.17)	0.586
Phosphorus (mmol/L), mean (SD)	1.77 (0.52)	1.89 (0.47)	0.389
PTH (pg/mL), mean (SD)	301 (151)	309 (186)	0.864
25(OH)vitamin D (µg/L), mean (SD)	16.1 (9.8)	21.2 (19.5)	0.286
Calcitriol supplementation, n (%)	16 (76.2)	17 (58.6)	0.196
Immunosuppressive medication, n (%)	4 (19.0)	5 (17.2)	0.870
anti-SARS-CoV-2-spike IgG after 1^st^ dose (BAU/mL), median (Q1, Q3)	56.7 (48.9, 101.7)	12.2 (6.2, 16.8)	<0.001
anti-SARS-CoV-2-spike IgG after 2^nd^ dose (BAU/mL), median (Q1, Q3)	1565.0 (1022.5, 2080.0)	635.5 (118.3, 1352.5)	0.001

spKt/V, single-pool Kt/V; CRP, C-reactive protein; PTH, parathyroid hormone.

^#^spKt/V given as the weekly mean in the week prior to vaccination.

Patients who reported a local reaction after injection showed a numerically higher median (Q1, Q3) anti-SARS-CoV-2 spike IgG concentration after the complete vaccination course [1210 (751.3, 1817.5) *vs* 763 (201.5, 1705) BAU/mL], but this difference was not statistically significant (p=0.245). The antibody concentration four weeks after the second vaccine injection was significantly higher in patients with documented hepatitis B seroconversion at baseline and detectable anti-HBs antibodies after earlier active hepatitis B vaccination (1440 [961, 2080] BAU/mL, n=23) compared with hepatitis B vaccination non-responders (308.5 [176.8, 1622.5] BAU/mL, n=12; p=0.035). Hemodialysis patients with documented immunity after hepatitis B infection (positive anti-HBc antibodies) were excluded from this analysis. Immunosuppressed patients (n=9) developed lower antibody concentration (592 [64.1, 1210.0] BAU/mL] compared to patients without additional immunosuppression (n=39; 1130 [576.0, 1800.0] BAU/mL; p=0.046). In a multiple linear regression analysis (**Table 3**) higher age (β: -14.1, 95% CI: -28.0, -0.2; p=0.046) and immunosuppression (β: -560.4, 95% CI: -1100.2, -20.7; p=0.042) were associated with a lower antibody concentration after the second vaccine dose, whereas calcitriol treatment was associated with an increased immune response (β: 488.1, 95% CI: 885.0, 91.2; p=0.017).

**Table 3:** Baseline predictors of the antibody concentration four weeks after the 2^nd^ vaccine dose.

Characteristic	B	95% CI	SE	P
Age (per year)	-14.1	-28.0, -0.2	6.9	0.046
Gender (male *vs* female)	-178.6	-588.1, 230.9	202.8	0.384
Dialysis vintage (per month)	0.4	-3.9, 4.7	2.1	0.855
Diabetes mellitus (yes *vs* no)	345.5	-92.0, 783.0	216.7	0.118
Calcitriol treatment (yes *vs* no)	488.1	91.2, 885.0	196.5	0.017
Immunosuppression (yes *vs* no)	-560.4	-1100.2, -20.7	267.3	0.042

B, regression coefficient (B); SE, standard error; 95% CI, 95% confidence interval of regression coefficient B.

During a follow-up of 90 days after the first vaccine dose only one patient was diagnosed with symptomatic SARS-CoV-2 infection. A 48-year-old woman was tested SARS-CoV-2 positive (PCR cycle threshold value 15.6) on the day of the second vaccination. She suffered from mild flu-like symptoms. At that time, she had already developed seroconversion after the first vaccine dose with an anti-SARS-CoV-2 spike IgG concentration of 49.7 BAU/mL. She did not receive the second vaccine dose and remained almost asymptomatic. One week later she had a PCR cycle threshold of 33.4. Four weeks after the diagnosis her anti-SARS-CoV-2 spike IgG concentration had increased to 2080 BAU/mL.

## Discussion

In our study, we present detailed data on the safety and immunogenicity of the mRNA-BNT162b2 SARS-CoV-2 vaccine in chronic hemodialysis patients. We found a good safety profile with a low rate of local and systemic side reactions and a high seroconversion rate of 97.9% in this cohort with a substantial antibody response after the complete vaccination course.

To our knowledge, no safety data of SARS-CoV-2 vaccines in hemodialysis patients have been published so far. Compared to the general population, a lower percentage of hemodialysis patients suffered from local reactions after the vaccine injections. In the pivotal trial with the same vaccine by Polack et al. (5), 70% to 80% of individuals reported local pain at the injection site, whereas approximately half as many patients in our study complained about this side effect. The participants´ age difference with an on average 15 years older cohort in our study may explain this finding, because an age-dependence with fewer local reactions in people aged >55 years compared with younger study participants has already been described in the general population (5). Importantly, no significant local hematoma occurred despite intramuscular injection during the hemodialysis session with full low-molecular weight heparin anticoagulation. Therefore, intramuscular vaccination in hemodialysis patients can be performed safely during the last 30 minutes of the hemodialysis treatment session. This enables sufficient observation after vaccination with adequate monitoring of vital signs, and no change in patients´ logistics and hemodialysis prescription is required. This approach is in accordance with the CDC general best practice guidelines for immunization in patients with bleeding disorders or taking warfarin in the general population (17).

Similarly, systemic reactions during the first week after the injection were found in only eight percent of the patients in our study, which is also less frequent than in the general population (5). Only 2.1% of patients reported severe muscle or joint pain, which were the only reactions that had numerically increased after the second injection compared to the first one. No patient reported a grade 4 local or systemic reaction requiring hospitalization. The incidence of local or systemic reactions in our study was also lower when compared to the second available mRNA vaccine mRNA-1273 (4).

Although the clinically meaningful antibody concentration cut-off value for definite seroprotection is unknown at present, the high seroconversion rate in our hemodialysis cohort is somehow surprising when compared to the response to other vaccines. After hepatitis B vaccination a seroconversion rate from 40% to 70% was detected in hemodialysis patients (8–11). Vaccination against the seasonal influenza and the 2009 pandemic influenza A virus H1N1 in hemodialysis patients resulted in seroconversion rates varying from 25% to 57% (18–22) with higher response rates using adjuvanted versus non-adjuvanted vaccines (23). In comparison to these findings, the strong immunogenicity with the mRNA-BNT162b2 SARS-CoV-2 vaccine found in our study is encouraging and hopefully translates into the prevention of clinically important outcomes such as severe COVID-19, hospitalization and death. Our results confirm the recently published high antibody response rates from 90% to 96% in three dialysis cohorts from Israel (24–26), and show a better vaccine response compared to 56 French SARS-CoV-2 infection-naïve hemodialysis patients (82%) (27). Vaccine safety data were reported in none of these studies. Nevertheless, the weak seroconversion rate of only 42% after the first vaccine dose in our study emphasizes that most of these vulnerable and high-risk patients are not protected after the first vaccine dose. Safety precautions and personal protective measures must be maintained at least until the second vaccine dose has been administered. Due to this delayed antibody response hemodialysis patients should be prioritized for rapid vaccination strategies, and the interval between first and second dose should not be extended.

The high rates of side reactions and good antibody responses with mRNA-based vaccines points to the possibility that they are more immunogenic compared to conventional vaccines. The mRNA vaccines have been shown to stimulate the production of neutralizing antibodies targeting the same epitopes in a manner similar to natural infection (28). In an attempt to explore a possible relationship between local reaction at the injection site and antibody response after the complete vaccination course, we determined the antibody concentration in patients with and without local reaction. Although patients who reported local pain had numerically higher concentrations than patients without, this difference did not reach statistical significance. Interestingly, patients with a documented seroconversion after prior active hepatitis vaccination with a second-generation recombinant hepatitis B vaccine had a significantly higher antibody response after the SARS-CoV-2 vaccination compared to non-responders. This finding supports the hypothesis that the vaccination response allows an integrated interpretation of the patients´ general immune competence. The fact that all but one hepatitis B vaccination non-responders seroconverted upon the mRNA-BNT162b2 SARS-CoV-2 vaccine points to a higher immunogenicity and efficacy of mRNA platform-based vaccines. Future mRNA-based hepatitis B vaccines may possibly lead to higher seroconversion rates in dialysis patients.

Age is a well-known key driver of vaccination response in hemodialysis patients with higher age being associated with a weaker antibody concentration as shown in the studies by Grupper at al (25). and Agur et al. (24), and substantiated in our study. Patients with seroconversion after the first vaccine dose were significantly younger than those without, and higher age was significantly associated with a lower antibody concentration four weeks after the complete vaccination course.

Active calcitriol treatment during vaccination was associated with a higher antibody concentration after the complete vaccination course. Most immune cells express vitamin D receptors (29). Additionally, dendritic cells, macrophages and monocytes convert the precursor 25(OH) vitamin D to active calcitriol using their own 1α-hydroxylase (30). Upon toll-like receptor-mediated activation and induced by increased calcitriol dendritic cells can migrate from the vaccination site to non-draining lymphoid organs, where they can stimulate antigen-specific T and B lymphocytes to produce a significant antibody response after vaccination (31, 32). Calcitriol stimulates the production of the Th2-like cytokines IL-4, IL-5, IL-10 and IL-13 supporting the humoral immune response (33–35).

Immunosuppressed patients had significantly lower anti-SARS-CoV-2 spike antibody concentrations, but still nearly all these patients (8/9) showed a seroconversion. Six out of these patients were treated with 5 mg prednisolone daily during the vaccination course, one patient with 5 mg prednisolone and 2 mg tacrolimus daily, one patient with 5 mg prednisolone and 150 mg azathioprine daily, and one patient had been treated with dexamethasone, bortezomib and thalidomide followed by autologous hematopoietic cell transplantation four months prior to vaccination and lenalidomide maintenance therapy during vaccination. The good response rate observed in immunosuppressed hemodialysis patients raises hope to a protective seroconversion with the mRNA-BNT162b2 SARS-CoV-2 vaccine in other patient groups with immunosuppression, although the response rate in patients with more profound immunosuppressive therapy, especially kidney transplant recipients has to be evaluated.

Certain limitations of our study must be acknowledged. The small cohort size is a limitation, but we included all patients consenting to vaccination in our centre without any selection criteria. Due to the uniform ethnic nature of our Caucasian hemodialysis cohort, we cannot generalize our findings to other ethnicities. Our results are limited to SARS-CoV-2 infection-naïve seronegative patients and the mRNA-BNT162b2 vaccine. Although neutralizing capacity cannot be directly derived from positive anti-SARS-CoV-2 spike IgG antibodies, the assay used in our study has a very good correlation with the microneutralization test. Furthermore, Speer et al. recently found positive anti-S1 IgG antibodies in 18% of 22 German hemodialysis patients (4/22) three weeks after the first vaccination, and in 82% (14/17) three weeks after the second vaccination using the mRNA BNT162b2 vaccine (36). The same number of patients had SARS-CoV-2 neutralizing antibodies (18% after 1st vaccination, 82% after 2^nd^ vaccination), a finding which is in line with the assumption that IgG antibodies directed against the S1 spike protein and RBD correspond to virus neutralizing antibodies. We were unable to determine the cellular immune response to vaccination (37, 38). Furthermore, we did not perform regular PCR-based screening of asymptomatic patients during the four weeks between first and second vaccination. Therefore, we cannot exclude the possibility of a positive antibody response or amplification of the vaccination response caused by an asymptomatic SARS-CoV-2 infection. However, whenever a patient presented with equivocal symptoms, antigen- and PCR-based testing was applied. In the case of a positive test result, all fellow patients of the same dialysis shift were tested at each session for one week.

As a strength of our work, all patients were serologically proven SARS-CoV-2 negative prior to vaccination. Therefore, we can exclude a booster effect of vaccination after undetected asymptomatic infection. Moreover, we assessed the safety profile of the mRNA-BNT162b2 SARS-CoV-2 vaccine in detail in chronic hemodialysis patients. So far, vaccine safety data have been missing in this patient group. Our approach, similar to the one used in the pivotal trial by Polack et al. (5) to evaluate side effects enables the comparison to general population data.

Some open questions remain: How long do hemodialysis patients maintain a significant vaccine-induced antibody concentration? Keeping in mind the rapid antibody waning after hepatitis B or influenza vaccination, a booster dose, possibly virus variant-specific, may be necessary. Is there a seroprotective cut-off value and what is the vaccine efficacy concerning the most important clinical outcomes of prevention of (severe) COVID-19, COVID-19-related hospitalization and intensive care treatment and COVID-19-related mortality? Do SARS-CoV-2-variants escape the vaccine-induced immune response in hemodialysis patients?

## Conclusion

In conclusion, the mRNA-BNT162b2 SARS-CoV-2 vaccine appears to be safe and well-tolerated in hemodialysis patients and shows a high immunogenicity in these patients. These data support the prioritization and rapid vaccination of this highly vulnerable patient cohort.

## Data Availability Statement

The original contributions presented in the study are included in the article/supplementary material. Further inquiries can be directed to the corresponding author.

## Ethics Statement

The studies involving human participants were reviewed and approved by Ethics committee of the Innsbruck Medical University. The patients/participants provided their written informed consent to participate in this study.

## Author Contributions

BM, MB, HU, TW, and KL designed the study and revised the article. TD, JS, AA-N and HS-M collected and interpreted data and revised the article. EZ designed the study, analyzed and interpreted data, drafted and revised the article. All authors contributed to the article and approved the submitted version.

## Conflict of Interest

The authors declare that the research was conducted in the absence of any commercial or financial relationships that could be construed as a potential conflict of interest.
